# Exploring prognostic genes in the immune microenvironment of acute myeloid leukemia via weighted gene co-expression network analysis

**DOI:** 10.1097/MD.0000000000044062

**Published:** 2025-10-17

**Authors:** Xiao Hu, Ying Chen, Huan Li, Shifeng Lou, Fengxia Bai

**Affiliations:** a Department of Hematology, The Second Affiliated Hospital, Chongqing Medical University, Chongqing, China; b Hematology Laboratory, The Second Affiliated Hospital, Chongqing Medical University, Chongqing, China.

**Keywords:** acute myeloid leukemia, biomarkers, bone marrow, differentially expressed genes, microenvironment, survival, weighted gene co-expression network analysis

## Abstract

**Background:**

Acute myeloid leukemia (AML) is a heterogeneous blood cancer that arises from transformed myeloid precursor cells in a compromised bone marrow microenvironment. This environment is essential for AML initiation, progression, and relapse. Alongside oncogenic changes in hematopoietic cells, immunological dysregulation also contributes to leukemogenesis. The present study is aimed to identify prognostic genes in stromal and immune cells associated with AML using the weighted gene co-expression network analysis (WGCNA).

**Methods:**

Gene expression profiles were retrieved from The Cancer Genome Atlas database, and immune and stromal cell scores were calculated using the ESTIMATE (Estimation of STromal and Immune cells in MAlignant Tumor tissues using Expression data) method. These scores helped identify differentially expressed genes (DEGs), which were then used to create gene clusters through WGCNA. To explore the functions of genes linked to AML subtypes, Gene Ontology and Kyoto Encyclopedia of Genes and Genomes enrichment analyses were performed. A protein–protein interaction network was developed to identify hub genes. The top 18 hub genes were identified using the cytoHubba plug-in in Cytoscape software, and survival analysis was conducted with the Gene Expression Profiling Interactive Analysis 2 online tool.

**Results:**

A total of 1097 DEGs were identified, with 601 being upregulated and 496 downregulated. WGCNA analysis indicated that the gray module, comprising 165 genes, had the strongest association with AML subtypes (Cor > 0.3; *P* < .05). Gene Ontology enrichment analysis demonstrated that the 18 identified hub genes were predominantly associated with neutrophil activation, immune response, secretory granule membrane, and pattern recognition receptor activity. Kyoto Encyclopedia of Genes and Genomes pathway enrichment analysis revealed that the DEGs were mainly involved in pathways related to phagosome, lysosome, tuberculosis, leishmaniasis, and neutrophil extracellular trap formation. Kaplan–Meier survival analysis of the top 18 hub genes indicated that ITGAM, IL10, and CD163 were significantly correlated with survival outcomes in AML.

**Conclusion:**

Key stromal and immune-related genes influencing AML patient outcomes were identified, highlighting their potential as therapeutic targets. These discoveries provide deeper insights into the molecular mechanisms driving AML pathogenesis and subtype differentiation.

## 1. Introduction

Acute myeloid leukemia (AML) is a malignant and highly heterogeneous disease characterized by the clonal expansion of undifferentiated myeloid precursors within hematopoietic progenitor of the bone marrow (BM).^[1,2]^ Although AML can occur at any age, the median age of diagnosis is 72, according to the Swedish Acute Leukemia Registry.^[3]^ The French–American–British classification originally identified 8 AML subtypes (M0–M7) based on cytochemical and morphological characteristics of malignant cells.^[4]^ In 2008, the World Health Organization updated the classification to integrate morphology, genetics, immunophenotype, and clinical features.^[5]^ This revision resulted in 6 major subtypes: AML with myelodysplasia-related features, AML with recurrent genetic abnormalities, therapy-related AML, AML not otherwise specified, myeloid proliferation associated with Down syndrome, and myeloid sarcoma.^[6,7]^ The emergence of advanced flow cytometry and next-generation sequencing has deepened our understanding of the molecular and genetic complexities of AML. It is now ascertained that the therapeutic response and prognosis of AML patients are significantly affected by molecular and cytogenetic abnormalities.^[8,9]^ The 2022 International Consensus Classification and the fifth edition of the World Health Organization classification updated and introduced new AML categories to better align with genetic and clinical data. While advances in personalized therapy have improved outcomes for many AML cases, refractory and relapsed AML remain common due to residual disease in the BM microenvironment.^[10–13]^ Therefore, more detailed cellular and molecular insights into AML pathogenesis within the BM are crucial for enhancing prevention, diagnosis, and personalized treatments. The BM microenvironment is a dynamic network consisting of stromal cells, hematopoietic progenitor cells, endothelial progenitors, mesenchymal progenitors, immune cells, and the extracellular matrix. These components, along with cell-released mediators like cytokines and growth factors, collectively support leukemogenesis.^[14–16]^ Both immune and stromal cells play critical roles in the development of hematologic malignancies.^[17–20]^ Recent years have seen significant advancements in immunotherapy for AML, with many therapies targeting stromal and immune-regulatory factors within malignant tissues.^[21–23]^

**Figure 1.**
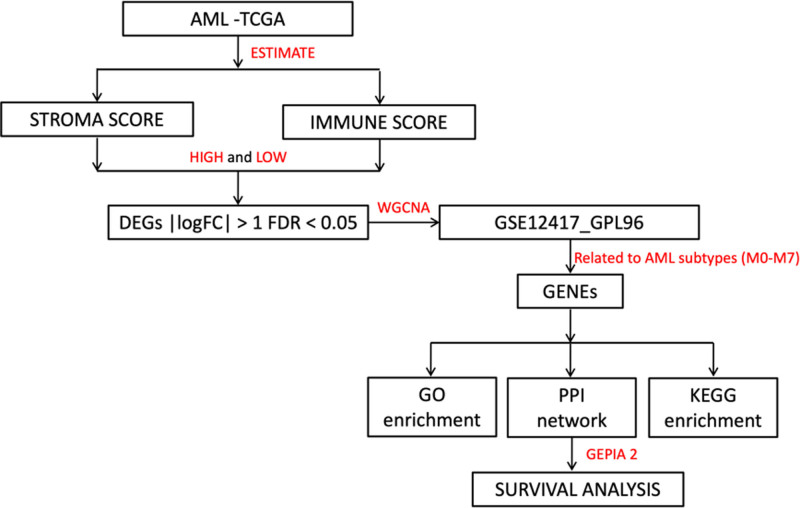
Workflow schema of the present study. AML =acute myeloid leukemia, DEGs = differentially expressed genes, FDR =false discovery rate, GEPIA2 = Gene Expression Profiling Interactive Analysis 2, GO = Gene Ontology, KEGG = Kyoto Encyclopedia of Genes and Genomes, PPI = protein–protein interaction, TCGA = The Cancer Genome Atlas, WGCNA = weighted gene co-expression network analysis.

Despite advances in transcriptomics and microarray bioinformatics, there remain few studies identifying potent biomarkers associated with AML.^[24,25]^ The ESTIMATE (Estimation of STromal and Immune cells in MAlignant Tumor tissues using Expression data) method utilizes gene expression signatures to predict immune and stromal cell infiltration in malignant tissues through single-sample gene set enrichment analysis.^[26]^ Recently, ESTIMATE was employed to investigate prognostic signatures in AML patients,^[27]^ with another study demonstrating that AML prognosis is linked to immune scores.^[28]^ Weighted gene co-expression network analysis (WGCNA) is a primary method for identifying clusters (or modules) of highly correlated genes.^[29]^ It enables to summarize these modules using either module eigengenes or intramodular hub genes, explore relationships between modules and external traits through eigengene network analysis, and calculate module membership scores. By constructing correlation networks, WGCNA also supports network-based gene screening, which can aid in the discovery of potential biomarkers or therapeutic targets. WGCNA is particularly valuable for identifying hub genes and co-expression patterns in pathological conditions.^[30,31]^ In gene networks, discovering a hub gene with numerous interactions with other genes is crucial for understanding gene regulation in biological processes and disease pathophysiology.^[32–34]^ WGCNA is a powerful tool for analyzing transcriptome data and identifying gene network signatures associated with complex diseases,^[35]^ including predicting AML prognosis.^[36,37]^ WGCNA complements other R-based network analysis tools by supporting both weighted and unweighted correlation networks. It functions as an exploratory and gene screening tool, enabling analysis of module structure, gene-module relationships, eigengene networks, and trait associations. WGCNA also aids in generating hypotheses, such as identifying disease-related modules for validation in independent datasets. In this study, whole transcriptome data from AML patients in The Cancer Genome Atlas (TCGA) were analyzed to calculate immune and stromal scores using the ESTIMATE algorithm. AML-related gene expression signatures were identified through WGCNA using data from the Gene Expression Omnibus (GEO). Hub genes and their associated gene sets underwent gene set enrichment analysis and Gene Ontology (GO) analysis to explore their functional roles in AML. Their prognostic relevance was validated using Gene Expression Profiling Interactive Analysis 2 (GEPIA2) with GEO data (Fig. 1). The goal was to identify key genes for predicting AML prognosis and stratifying patient risk.

## 2. Materials and methods

### 2.1. TCGA data collection and processing

RNA sequencing data from 151 adult AML patients, along with their corresponding clinical information, were retrieved from the TCGA database (https://portal.gdc.cancer.gov/; Table 1).

**Table 1:** Clinical data of the CN-AML subjects included in the analysis.

Variable	TCGA dataset
Subjects (n)	151
Age (range), yr	56 (21–88)
<60 yr n (%)	76 (51%)
≥60 yr n (%)	75 (49%)
AML Subtype	CN-AML
Sample source n (%)	
Bone marrow	151 (100%)
OS *median (Range)*, d	366 (28–2861)
Types n (%)	
M0	16 (11%)
M1	35 (23%)
M2	38 (25%)
M3	15 (10%)
M4	29 (19%)
M5	15 (10%)
M6	2 (1%)
M7	1 (1%)
Not classified	1 (1%)
Status n (%)	
Alive	54 (36%)
Death	97 (64%)

CN-AML = cytogenetically normal acute myeloid leukemia, OS = overall survival, TCGA = The Cancer Genome Atlas.

Only samples with complete clinical information and available RNA-seq data were included. Genes with low expression levels (e.g., counts per million < 1 in more than 50% of samples) were excluded to reduce noise and ensure data quality. Normalization and filtering steps were performed prior to downstream analysis to maintain consistency and reliability.

Datasets were merged into a single matrix, and the data was processed by converting them to official gene symbols using a Perl script. Additionally, messenger ribonucleic acid (mRNA) transcript identifiers were converted to gene symbols via a Perl script. The ESTIMATE method was then applied to calculate stromal and immune scores. Yoshihara et al^[26]^ proposed a new calculation method called ESTIMATE, wherein the algorithm estimates and obtains stromal and immune cell scores using cancer gene expression data. This score can then predict the infiltration levels of stromal and immune cells in the tumor microenvironment. ESTIMATE was chosen due to its robust performance and widespread use across various cancer types. Despite its strengths, we acknowledge that ESTIMATE is limited by its reliance on fixed gene signatures, which may not fully capture the heterogeneity of immune and stromal cell populations.

### 2.2. Screening of differentially expressed genes (DEGs)

AML patients were categorized into high-score and low-score groups based on their stromal and immune scores. In the Empirical Analysis of Digital Gene Expression Data in R (edgeR), differentially expressed gene (DEGs) were defined as those with |log_2_ (fold change)| ≥ 1 and a false discovery rate < 0.05.^[38,39]^ Only DEGs that were common to both immune and stromal score groups were used for further analysis.

### 2.3. Weighted gene co-expression network analysis (WGCNA)

The DEGs were clustered based on immune and stromal scores using the WGCNA package. The soft-thresholding power (β) was determined using the scale-free topology criterion in WGCNA, selecting the lowest power at which the scale-free fit index (*R*²) exceeded 0.85. A power of 9 and a correlation coefficient threshold of 0.8 were ultimately chosen. To ensure robustness, a sensitivity analysis was conducted across a range of powers, confirming consistent module detection. Sample clustering was performed using the “FlashClust” function in *R*, and the “pickSoftThreshold” function was used to refine the power selection. Genes most strongly correlated with AML subtypes were selected for downstream analysis.

### 2.4. GO and KEGG (Kyoto Encyclopedia of Genes and Genomes) enrichment analyses of DEGs

To evaluate the potential functions of genes associated with AML subtypes, Kyoto Encyclopedia of Genes and Genomes (KEGG) and GO enrichment analyses were performed using the “clusterProfiler” package, with a significance cutoff set at *P* < .05. GO enrichment analysis was conducted for biological process, cellular component and molecular function.

### 2.5. Protein–protein interaction (PPI) network construction and analysis of hub gene co-expression

A protein–protein interaction (PPI) network for hub genes associated with AML subtypes was constructed using the STRING 11.0 database (https://string-db.org/), with a confidence score threshold of ≥0.4. The network was visualized using Cytoscape software, and the Cytoscape plug-in cytoHubba was employed to identify the top 18 hub genes with the highest number of connections.

### 2.6. Analyzing survival and validating hub genes

Survival analysis was performed using the GEPIA2 module (http://gepia2.cancer-pku.cn/#index) to evaluate the overall survival (OS) of relevant mRNAs in AML. Kaplan–Meier curves were generated by dividing patients into high and low expression groups based on the median (50th percentile) gene expression level. Statistical significance was assessed using the log-rank test, and hazard ratios (HR) with 95% confidence intervals were reported. These details are now included in the revised Methods section for clarity.

## 3. Results

### 3.1. DEGs identification from stromal/immune scores of AML

**Figure 2.**
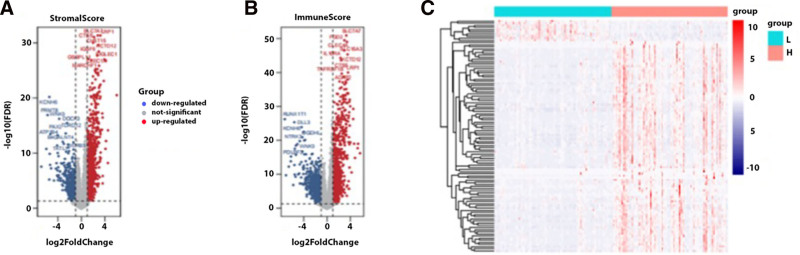
Volcano plots (A, B) and heatmap (C) of DEGs. A total of 1798 DEGs were identified based on stromal scores (838 upregulated, 960 downregulated) and 1592 DEGs based on immune scores (888 upregulated, 704 downregulated). Integrated bioinformatics analysis identified 1097 DEGs as significant for the divergent phenotype (high vs low stromal/immune score groups), with 601 upregulated and 496 downregulated DEGs. Analyses then focused on these 1097 DEGs. DEGs = differentially expressed genes.

Transcriptomic data from 151 AML patients were retrieved from TCGA and analyzed to explore associations between transcript profiles and stromal/immune scores, with cutoff values set at |log_2_ (fold change)| ≥ 1 and false discovery rate < 0.05. This analysis identified 1798 DEGs based on stromal scores (838 upregulated and 960 downregulated) and 1592 DEGs based on immune scores (888 upregulated and 704 downregulated; Fig. 2A). An integrated bioinformatics analysis then revealed 1097 DEGs as highly significant for the divergent phenotype (high vs low stromal/immune score groups), with 601 upregulated and 496 downregulated DEGs. Subsequent analyses focused on these 1097 DEGs (Fig. 2B).

### 3.2. Gene clustering by WGCNA

**Figure 3.**
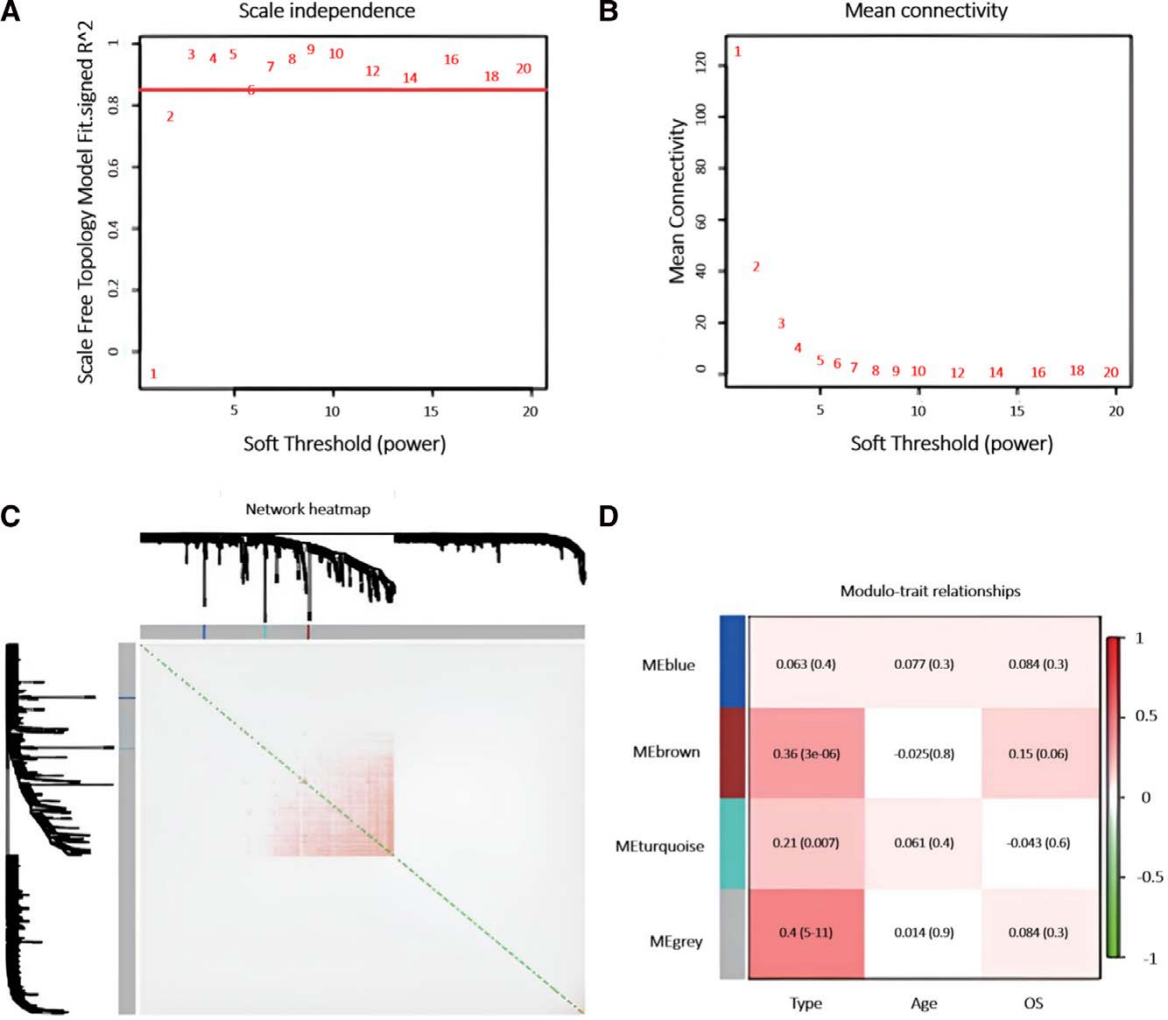
Clustering of DEGs using WGCNA. Analysis of the scale-free fit index (A) and mean connectivity (B) across different soft-thresholding powers. (C) Heatmap displaying network overlap within co-expression modules; deeper red hues indicate stronger overlap among functional modules. (D) Heatmap of module-trait correlations; deeper red hues indicate a stronger association with AML subtypes, age, and OS. AML = acute myeloid leukemia, DEGs = differentially expressed genes, OS = overall survival, WGCNA = weighted gene co-expression network analysis.

To refine the WGCNA model analysis, we plotted the relationship between the soft threshold correlation coefficient and the mean gene connection coefficient (Fig. 3A). A soft-thresholding power of 9 was chosen based on a correlation coefficient threshold of 0.8 (Fig. 3B). WGCNA identified 4 distinct co-expression modules (Fig. 3C). Module-trait correlation analysis revealed that several modules were associated with AML subtypes M0–M7, with the gray module showing the strongest correlation. We selected 165 genes from the gray module, having Cor > 0.3 and *P* < .05, for further analysis (Fig. 3D).

### 3.3. GO and KEGG enrichment analysis

**Figure 4.**
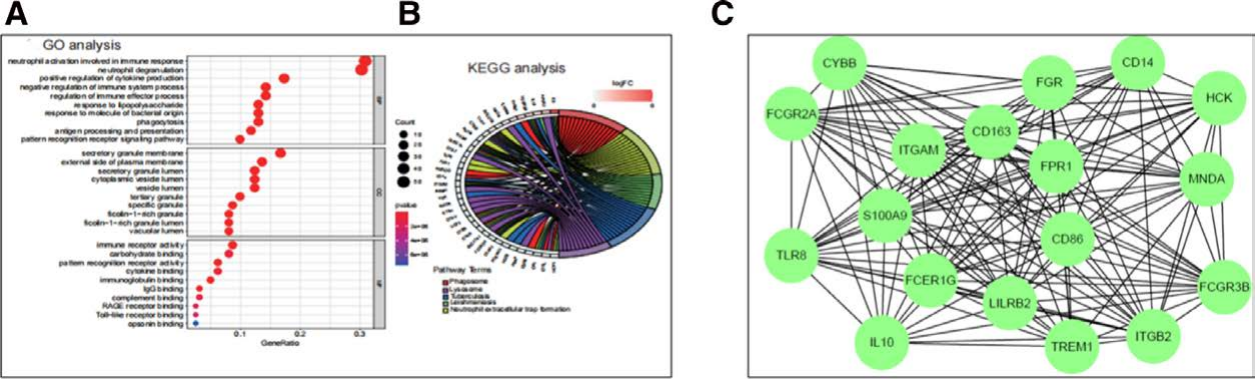
GO and KEGG pathway enrichment analysis of mRNA. In the GO enrichment analysis (A), BP branch DEGs are linked to neutrophil activation, degranulation, cytokine production, and antigen processing/presentation. In the CC branch, DEGs are associated with tertiary granules, secretory granule membranes, and cytoplasmic vesicle lumens. MF branch DEGs relate to pattern recognition receptor activity, immunoreceptor activity, and immunoglobulin binding. KEGG pathway analysis identified 165 genes involved in phagosome, lysosome, tuberculosis, leishmaniasis, and neutrophil extracellular trap formation (B). The STRING database was used to construct the PPI network of AML DEGs (C). Of 165 genes, 144 were mapped, forming a network with 144 nodes and 1024 edges. Using CytoHubba Degree algorithm, 18 top hub genes (ITGAM, LILRB2, TLR8, CD86, IL10, CD14, FCGR2A, FCGR3B, FCER1G, ITGB2, CD163, MNDA, CYBB, HCK, S100A9, TREM1, FGR, and FPR1) were identified. AML = acute myeloid leukemia, BP = biological process, CC = cellular component, DEGs = differentially expressed genes, GO = Gene Ontology, KEGG = Kyoto Encyclopedia of Genes and Genomes, MF = molecular function, mRNA = messenger ribonucleic acid, PPI = protein–protein interaction.

In the biological process branch of the GO enrichment analysis (Fig. 4A), the most significantly enriched DEGs are associated with neutrophil activation, neutrophil degranulation, positive regulation of cytokine production, and antigen processing and presentation. In the cellular component branch, the enriched DEGs are related to tertiary granules, secretory granule membranes, cytoplasmic vesicle lumen, and secretory granule lumen. In the molecular function branch, the DEGs are linked to pattern recognition receptor activity, immunoreceptor activity, and immunoglobulin binding. The KEGG pathway analysis identified that 165 genes are functionally associated with phagosome, lysosome, tuberculosis, leishmaniasis, and neutrophil extracellular trap formation (Fig. 4B).

### 3.4. PPI web and hub genes co-expression

Data from the STRING online database were used to construct the PPI network of DEGs in AML. Out of the 165 genes, 144 were mapped onto the PPI network, resulting in a network with 144 nodes and 1024 edges. Additionally, 18 top hub genes (ITGAM, LILRB2, TLR8, CD86, IL10, CD14, FCGR2A, FCGR3B, FCER1G, ITGB2, CD163, MNDA, CYBB, HCK, S100A9, TREM1, FGR, and FPR1) were identified using the Degree algorithm in CytoHubba software (Fig. 4C).

The Degree algorithm in CytoHubba – a plug-in for Cytoscape designed to identify key nodes such as hub genes or proteins within biological networks – is one of the simplest centrality measures available. It calculates the degree of each node, which refers to the number of direct connections (edges) a node has with other nodes in the network. Nodes with a higher number of connections receive a higher degree score, allowing CytoHubba to rank them based on their level of connectivity. The selection of these 18 hub genes reflects both their high connectivity within the PPI network and their potential biological significance.

### 3.5. Analyzing survival and validating gene expression

**Figure 5.**
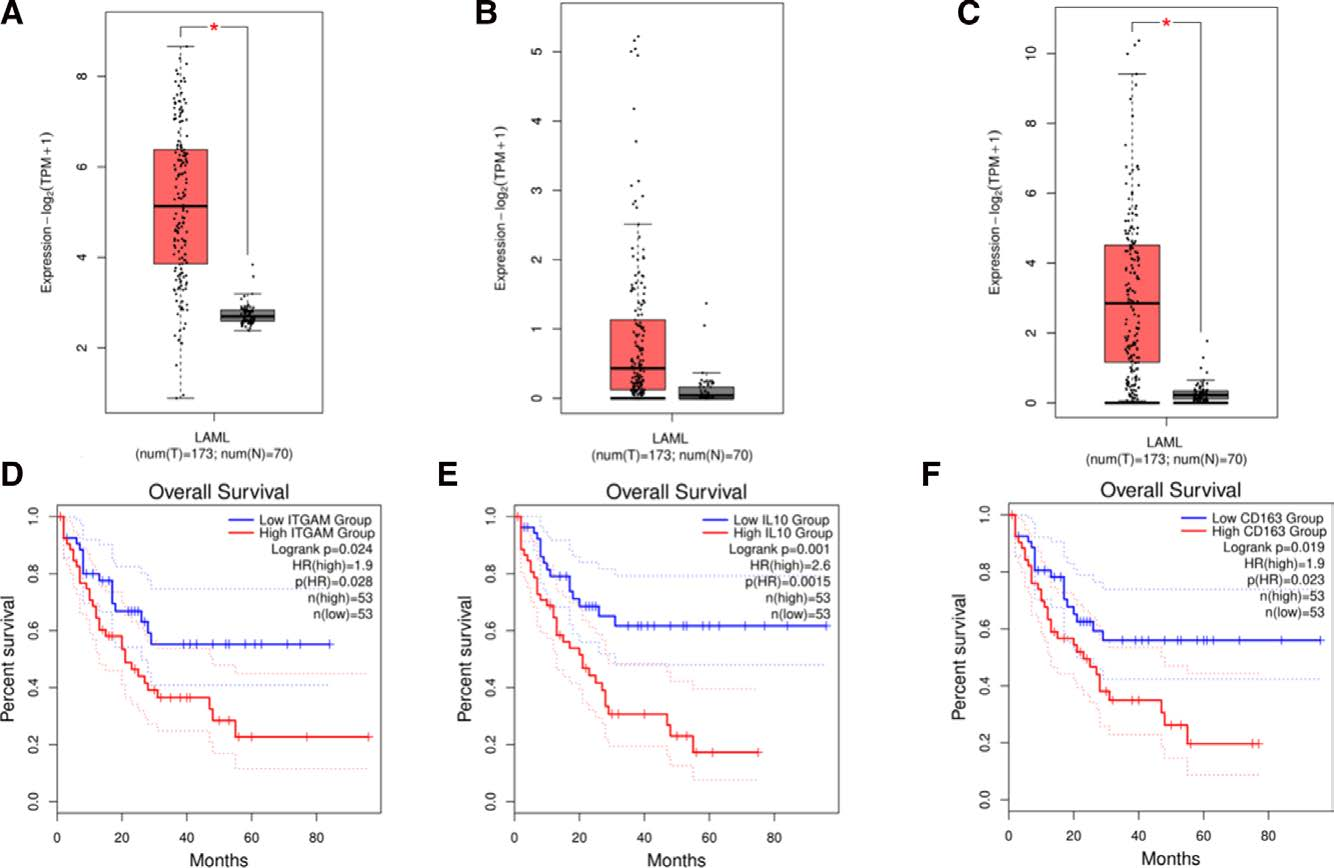
The GEPIA2 database validated the expression levels of the top 18 hub genes in normal versus AML tissues. Only ITGAM and CD163 showed significant differences (*P* < .05) between normal and AML tissues, according to TCGA and Genotype-Tissue Expression Project data. Survival analysis of these genes revealed that ITGAM (HR = 1.9, log-rank *P* = .024), IL10 (HR = 2.6, log-rank *P* = .001), and CD163 (HR = 1.9, log-rank *P* = .019) were significantly associated with OS in AML, with high mRNA levels of these genes correlating with poorer OS (A–F). AML = acute myeloid leukemia, GEPIA2 = Gene Expression Profiling Interactive Analysis 2, HR = hazard ratio, mRNA = messenger ribonucleic acid, OS = overall survival, TCGA = The Cancer Genome Atlas.

The expression levels of the top 18 hub genes in normal versus AML tissues were validated using the GEPIA2 database (Fig. 5A–C). Among these, only ITGAM and CD163 showed significant differences (*P* < .05) between normal and AML tissues, based on data from TCGA and the Genotype-Tissue Expression Project. Additionally, survival data for these 18 AML-associated genes were analyzed using the GEPIA2 database, with survival plots shown in Figure 5D–F. Three genes ITGAM (HR = 1.9, Log-rank *P* = .024), IL10 (HR = 2.6, Log-rank *P* = .001), and CD163 (HR = 1.9, Log-rank *P* = .019), were significantly associated with OS in AML patients. Notably, high mRNA levels of ITGAM, IL10, and CD163 correlated with poorer OS in AML patients.

## 4. Discussion

AML is a malignant and highly heterogeneous blood cancer in which the BM microenvironment plays a significant role in disease pathophysiology.^[40]^ Currently, there are no universally recognized biomarkers for the early diagnosis, prognosis and effective treatment of AML to improve the OS of patients. In AML, only using a combination of potential biomarkers is beneficial for early risk assessment, accurate diagnosis, prognosis evaluation, and monitoring of the disease.^[41–45]^ In this study, we utilized ESTIMATE and WGCNA to identify key BM microenvironment-associated genes that could potentially serve as predictors of patient outcomes, using a well-defined cohort of AML patients. To our knowledge, this is the first study to identify prognostic survival markers through these bioinformatic methods. We examined stromal and immune-related genes in AML patients using data from the TCGA and GEO databases. Stromal and immune scores were fundamental in identifying DEGs, which were used to construct a WGCNA gene network. These DEGs were further analyzed through GO and KEGG pathway enrichment analyses, along with PPI network predictions. The cytoHubba tool was used to identify the top 18 hub genes, and GEPIA2 was employed for survival analysis. First, data obtained from the TCGA database facilitated the calculation of stromal and immune scores using the ESTIMATE method. This approach is biologically grounded, as stromal and immune cell types are integral components of the BM and play a significant role in the development of malignancy and resistance to therapy.^[46]^ Importantly, these stromal and immune scores were effective in identifying DEGs between low- and high-score groups. Although ESTIMATE has proven useful for identifying potential biomarkers, it is important to note that the algorithm was originally developed and validated using solid tumor data, where stromal and immune infiltration exhibit distinct spatial patterns. Moreover, its accuracy depends on predefined gene signatures for stromal and immune components, which may not fully reflect patient- or disease-specific variability in gene expression. Using WGCNA, gene co-expression networks were constructed based on the GSE12417 microarray dataset, revealing 4 key gene co-expression modules associated with AML subtypes. Beyond the critical gray module linked to AML subtypes, additional co-expression modules – turquoise, brown, and blue – were identified for each AML subtype. The enriched DEGs were primarily associated with neutrophil activation in immune responses and neutrophil degranulation, secretory granule membranes, and tertiary granules, as well as pattern recognition receptor activity. KEGG pathway analysis indicated that DEGs were also enriched in pathways related to the phagosome, lysosome, tuberculosis, leishmaniasis, and neutrophil extracellular trap formation. These findings align with previous research demonstrating the involvement of the BM immune system in AML pathogenesis. Fu et al^[47]^ reported that the DEGs were significantly enriched in biological processes related to cell activation involved in immune response, cytokine production, and leukocyte migration. Key pathways identified included the phagosome, antigen processing and presentation, cell adhesion molecules, cytokine–cytokine receptor interaction, Fc gamma R-mediated phagocytosis, B-cell receptor signaling, and chemokine signaling pathways, all of which are closely associated with immune responses during antitumor activity. Interestingly, Zhong et al^[48]^ recently identified 2 distinct molecular subtypes based on multi-omics analysis, characterized by high and low neutrophil extracellular traps (NET) scores. The low NET score subgroup showed enhanced infiltration of immune effector cells, indicating a more active immune environment. In contrast, the high NET score subtype was marked by a greater presence of monocytes and M2 macrophages, along with elevated expression of immune checkpoint genes, reflecting an immunosuppressive microenvironment. These features were associated with a significantly poorer prognosis, underscoring the link between NET-associated immunosuppression and adverse clinical outcomes.

Recently, the significance of these immune-related pathways in AML has been recognized, prompting the development of targeted immunotherapies.^[49,50]^ In this study we use the STRING database to develop a PPI network for DEGs in AML. A total of 144 genes were mapped onto this network, with the top 18 hub genes actively interacting, as shown in gene co-expression predictions. In survival analysis of these hub genes, 3 (ITGAM, IL-10, and CD163) were significantly associated with OS in AML patients. Specifically, high mRNA expression levels of these genes correlated with lower survival rates. Bachli et al^[51]^ demonstrated that the functional properties of CD163 are retained on malignant cells, revealing that M4/M5 blast cells can internalize Hb-Hp through a CD163-mediated process. Collectively, their findings suggest that CD163 could serve as a promising target for therapeutic intervention. Notably, CD163 does not seem to be released from leukemic blasts in noninflammatory conditions, which lowers the risk of off-target side effects caused by competitive binding of potential therapeutic ligands to non-membrane-bound CD163. ITGAM, encoding the integrin αM component of the Mac-1 complex (CD11b), is involved in leukocyte adhesion, migration, and phagocytosis in monocytes, macrophages, neutrophils, and dendritic cells.^[52,53]^ It has been reported that ITGAM may serve as a promising prognostic biomarker and therapeutic target for AML.^[54]^ Elevated ITGAM levels in AML are linked to poor survival outcomes. Silencing ITGAM decreases AML cell viability and triggers apoptosis by disrupting cell cycle progression, likely by inhibiting the activation of the MAPK pathway. CD11b plays a crucial role in clearing opsonized apoptotic cells and immune complexes, thereby mitigating inflammatory responses.^[55]^ Previous studies have linked ITGAM expression to immune suppression in AML.^[56]^ IL-10, an anti-inflammatory cytokine, is a critical regulator of immune responses, including angiogenesis and cancer invasiveness.^[57,58]^ IL-10 polymorphisms have also been associated with AML.^[59]^ Several transcriptomic studies have explored gene expression patterns and immune-related signatures in AML, providing important insights into disease pathogenesis and potential therapeutic targets. For instance, Yu et al^[60]^ constructed an immune infiltration-related gene model that identify 8 hub genes with good risk stratification and predictive prognosis for AML, while Zhang et al^[61]^ identified a set of high-risk genes that may be used as prognostic and therapeutic markers for AML patients. In addition, significant use of the ML algorithms in constructing and validating the prognostic models in AML was demonstrated. Although our study used extensive bioinformatics and machine learning methods to identify the hub genes in AML, their experimental validations using knockout/-in methods would strengthen our findings. Consistent with these findings, our study also highlights the enrichment of immune-related pathways such as cytokine–cytokine receptor interaction, phagosome formation, and antigen processing and presentation.

However, our study extends previous work by integrating WGCNA and CytoHubba to construct a systems-level understanding of gene co-expression and protein–protein interaction networks. This approach enabled us to identify 18 key hub genes with high connectivity, several of which (ITGAM, LILRB2, TLR8, CD86, IL10, CD14, FCGR2A, FCGR3B, FCER1G, ITGB2, CD163, MNDA, CYBB, HCK, S100A9, TREM1, FGR, and FPR1) have not been previously reported in the context of AML. Additionally, by incorporating immune infiltration analyses and linking hub gene expression to specific immune cell populations and checkpoint molecules, our study provides a more comprehensive view of the immunosuppressive landscape in AML.

These integrative findings not only reinforce known immune dysfunctions in AML but also suggest novel candidates for further functional validation and potential therapeutic targeting.Our results align with previous research indicating that ITGAM and IL-10 are linked to AML subtypes characterized by cell survival and proliferation.^[56,62]^ CD163, expressed primarily by blast cells in AML subtypes M4 and M5 rather than other hematopoietic progenitors, belongs to the cysteine-rich scavenger receptor family.^[63,64]^ Its lineage-specific expression in certain AML blast cells suggests CD163 could be a promising therapeutic target for specific AML cases. Our findings indicate that ITGAM, IL-10, and CD163 hold potential as therapeutic targets and could be crucial in developing AML subtype-specific treatments. Nevertheless, the present study has some limitations. The sample size of AML cases in the database may be insufficient. Although gene data from 151 AML patients were included, further evaluation is needed to determine whether this sample size is sufficient to ensure the accuracy and generalizability of the findings. Moreover, patient heterogeneity and technical variations could influence the results. Despite the promising insights gained from this study, several limitations should be acknowledged. First, the relatively small sample size may limit the statistical power and robustness of the findings. Second, the lack of validation using external datasets restricts our ability to confirm the reproducibility and generalizability of the identified hub genes and enriched pathways across diverse AML populations.

To address these limitations, future studies should aim to validate these results in larger, independent cohorts, including those with different AML subtypes and clinical backgrounds. In addition, functional assays and experimental models will be essential to further investigate the biological roles of the identified hub genes and their potential as diagnostic or therapeutic targets.

## 5. Conclusion

Identifying key genes that can predict AML prognosis and stratify patients according to risk is critically important. In this study, DEGs were identified in a well-characterized cohort of AML patients and analyzed for their prognostic value. The stromal and immune-related genes found to be significantly associated with AML could have practical clinical applications, either as biomarkers for patient prognosis or as potential new drug targets to improve therapeutic outcomes in AML patients.

## Author contributions

**Conceptualization:** Ying Chen.

**Data curation:** Huan Li.

**Formal analysis:** Xiao Hu.

**Investigation:** Shifeng Lou.

**Methodology:** Fengxia Bai.
